# Effects of feed intake on the diversity and population density of homoacetogens in the large intestine of pigs

**DOI:** 10.5713/ajas.18.0512

**Published:** 2019-02-14

**Authors:** Hiroki Matsui, Ayumi Mimura, Sakiko Maekawa, Tomomi Ban-Tokuda

**Affiliations:** 1 Graduate School of Bioresources, Mie University, Tsu, Mie 514-8507, Japan; 2 Graduate School of Bioagricultural Sciences, Nagoya University, Chikusa, Nagoya 464-8601, Japan

**Keywords:** Feed Intake, Formyltetrahydrofolate Synthetase (FTHFS) Gene (*FHS*), Homoacetogen, Large Intestine, Pig

## Abstract

**Objective:**

Homoacetogens play important roles in the production of acetate in the large intestine of monogastric mammals. However, their diversity in the porcine large intestine is still unknown. Marker gene analysis was performed to assess the effects of energy level on the diversity and population densities of homoacetogens in porcine feces.

**Methods:**

Crossbred pigs were fed high or low energy-level diets. The high-intake (HI) diet was sufficient to allow a daily gain of 1.2 kg. The low-intake (LI) diet provided 0.6 times the amount of energy as the HI diet. Genetic diversity was analyzed using formyltetrahydrofolate synthetase gene (*FHS*) clone libraries derived from fecal DNA samples. *FHS* DNA copy numbers were quantified using real-time polymerase chain reaction.

**Results:**

A wide variety of *FHS* sequences was recovered from animals in both treatments. No differences in *FHS* clone libraries between the HI and LI groups were found. During the experimental period, no significant differences in the proportion of *FHS* copy numbers were observed between the two treatment groups.

**Conclusion:**

This is the first reported molecular diversity analysis using specific homoacetogen marker genes from the large intestines of pigs. There was no observable effect of feed intake on acetogen diversity.

## INTRODUCTION

Mammalian animals harbor an enormously diverse and dense microbiota in their gastrointestinal tract (GIT), particularly in the large intestine. This mutualistic microbiota plays an important role in determining animal nutrition and health. Dietary components that have not been digested in the stomach and small intestine, primarily indigestible carbohydrates, enter the large intestine and provide fermentation substrata for gut microbes. Short-chain fatty acids (SCFAs) produced during fermentation are transported across the epithelial cells of the large intestine and utilized as energy sources by the animal [1]. Fermentation by microbes in the GIT is important for energy homeostasis [2]. In pigs, SCFAs produced in the large intestine supply up to 30% of the energy required for maintenance [3]. Thus, SCFAs in the porcine large intestine are important energy sources and can have large effects on productivity.

In the large intestine of monogastric mammals and in the rumen of ruminants, homo acetogenic bacteria (acetogens) produce acetate by reducing CO_2_ using H_2_ via a pathway known as reductive acetogenesis [4–7]. A previous *in vitro* experiment using ^13^CO_2_ found that acetogens compete with methanogens for H_2_ in the large intestine of pigs [4]. Gases such as CO_2_, hydrogen, methane, and ammonia are produced by fermentation. Production of these gases is lower in the hind gut of monogastric animals than in the rumen of ruminants. Several studies have demonstrated that this disparity in methane production between monogastric species and ruminants is due to the increased production of propionate using hydrogen during reductive acetogenesis in monogastric species [5,8,9]. Thus, acetogens are vital for energy homeostasis in the large intestines of host animals. In our recent publication, the diversity of methanogens in the large intestines of pigs varied significantly in animals fed high intake (HI) diets relative to those fed low intake (LI) diets [10]. Therefore, we hypothesized that the diversity of acetogens might also differ between animals fed HI and LI diets.

Drake et al [11] pointed out that the development of 16S ribosomal RNA gene (16S rDNA) oligonucleotide primers that exclusively target all known acetogens is impossible, as their 16S rDNA sequences are not monophyletic and are often very closely related to non-acetogen taxa. Formyltetrahydrofolate synthetase (FTHFS) is a key enzyme in acetogenesis encoded by the *FHS* gene. Oligonucleotide primers that amplify the partial gene sequence encoding the enzyme gene have been developed and applied in acetogen diversity analyses [12]. Acetogen diversity has been successfully assessed in the rumen [13,14], human large intestine [7], and ostrich ceca [15] using *FHS* gene clone libraries. However, little is known about the diversity and population density of acetogens in the porcine large intestine.

In this study, pigs were fed 2 different diets each with a different intake level. We examined the effect of intake level on acetogen diversity and population size in pig feces. The *FHS* clone libraries were used to assess acetogen diversity. Real-time polymerase chain reaction (PCR) targeting the *FHS* gene was employed to quantify acetogen population densities.

## MATERIALS AND METHODS

### Animals and diets

Animals were handled according to the guidelines of Mie University. Eight crossbred pigs (Camborough×Duroc; average body weight 44.5±4.5 kg) were used in the study. After a 1-wk adaptation period, the animals were fed for 8 wks. The animals were individually caged during the experiment. Feed intake and body weight was recorded weekly.

The ingredients and chemical compositions of each diet are shown in Table 1. Both diets were mush-type diets. The animals were equally divided into 2 dietary groups, with each diet varying in its energy content based on differing provisioning rates. In the HI diet group, animals were fed a diet resulting in a daily gain of 1.2 kg. The LI diet provided 0.6 times the amount of energy that satisfied daily gain of 1.2 kg. Dietary provisioning rates were calculated based on the animals’ weights and according to the Japanese Feeding Standard for Swine [16]. Animals were fed at 09:00 and 16:00 each day.

### Sample collection and DNA extraction

Fecal samples were collected manually from the rectum (by gloved hands) at 09:00 on the first day of weeks 0, 4, and 8. Fecal samples were put in plastic bags and immediately placed on ice. The samples were then transferred to the laboratory where they were stored at −80°C before analysis.

Frozen fecal samples were thawed at 25°C. Total DNA was extracted from 0.25 g of each fecal sample using the UltraClean Fecal DNA Isolation kit (MO BIO Laboratories, Inc., Carlsbad, CA, USA). For the construction of clone libraries, the concentrations of DNA samples from each animal were adjusted to 15 ng/μL. The extracted DNA was stored at −25°C until analysis.

### Construction of *FHS* clone libraries and diversity analyses

DNA samples taken on week 8 were used to construct clone libraries. DNA solutions from each animal in each treatment were combined in equal parts and used as template for PCR. The *FHS* clone libraries were constructed following the methods of Matsui et al [14].

Sequences from the cloned DNA fragments were analyzed as described elsewhere [17]. The DNA sequences were used as search queries in BlastX [18]. Operational taxonomic units (OTUs), coverage, the Shannon-Wiener index (*H*′), and Chao1 were calculated using the DOTUR program [19]. Sequences were assigned to individual OTUs based on a 98% amino acid sequence similarity criterion [20]. The amino acid sequences were aligned using ClustalX ver. 2.0 [21], and phylogenetic trees were constructed using the neighbor-joining method [22]. Bootstrapping (1,000 resamplings) was used to estimate the confidence of branch patterns. The statistical significance of differences between the clone libraries was evaluated using the webLIBSHUFF program (http://libshuff.mib.uga.edu) [23].

Forty amino acid residues within the deduced amino acid sequence of *FHS* were used to calculate the homoacetogen similarity (HS) scores for *FHS* sequences. Sequences were then extracted from the alignment file and calculated using the methods described by Henderson et al [24]. Sequences with high HS scores (≥80%) were identified as possible homoacetogens.

### Real-time polymerase chain reaction assays

The DNA copy number of *FHS* and the DNA copy number of 16S ribosomal RNA (16S rDNA) of the total bacteria found in fecal samples were quantified using real-time PCR. All real-time PCR assays were performed using an ABI Prism 7000 Sequence Detection System (Applied Biosystems, Foster City, CA, USA) and SYBR green PCR master mix (Applied Biosystems, USA). The reaction mixture (25 μL) consisted of 1 μL of template, 12.5 μL of SYBR green master mix, and sterilized Milli-Q water. A primer pair, FTHFS for (5′-TTYAC WGGHGAYTTCCATGC-3′) and FTHFS rev (5′-GTATT GDGTYTTRGCCATACA-3′) was used in *FHS* analysis [25]. Assays were carried out using the following cycle conditions: 1 cycle at 50°C for 2 min, 1 cycle at 95°C for 2 min to allow initial denaturation, 40 cycles at 95°C for 15 s, and 60°C for 1 min to allow primer annealing and product elongation. Amplicon specificity was determined via dissociation curve analysis of PCR end products by increasing the temperature at a rate of 1°C/30 s from 60°C to 95°C. For *FHS*, standard DNA was prepared using an *FHS* gene fragment from *Blautia producta*.

Quantification of 16S rDNA was performed following the methods of Guo et al [26]. A primer pair, 530f (5′-GTGCCAG CMGCCGCGG-3′) and 920r (5′-GTCAATTCCTTTGAG TTT-3′) was used in rDNA assays. Standard DNA was prepared using a 16S rDNA fragment from *Escherichia coli*. The number of copies of *FHS* was expressed as the number of copies relative to the total number of bacteria.

### Nucleotide sequence accession numbers

All nucleic acid sequences obtained in this study were deposited in the DNA Data Bank of Japan, European Molecular Biology Laboratory, and GenBank databases under accession numbers AB623795–AB623894.

### Statistical analysis

Differences in the measured parameters between treatments were compared using unpaired *t*-test. Differences in mean measured parameters between weeks were compared using paired t-test. All statistical analyses were performed using a commercially available computer program (StatView; SAS Institute Inc., Cary, NC, USA). Differences were considered significant at p<0.05.

## RESULTS

### *FHS* gene diversity in porcine fecal material

The nucleotide sequence of the cloned DNA fragment was 1,095 to 1,104 bp in length. The deduced amino acid residues were 365 to 370 bp in length.

A total of 50 clones were randomly selected and analyzed for each library. Clones in the *FHS* library identified based on the analysis of material from the HI group were classified into 17 OTUs, with 20 OTUs being classified based on material from the LI group. The coverage of the *FHS* libraries derived from samples in the HI treatment and the LI treatment were 84% and 82%, respectively. The *H*′ of the *FHS* libraries derived from samples from the HI and LI treatments were 2.532 and 2.749, respectively. The Chao-1 estimation of HI and LI treatment were 31 and 27 species, respectively. No statistical differences were detected between the *FHS* clone libraries derived from the HI and LI treatments (data not shown).

**Figure 1:**
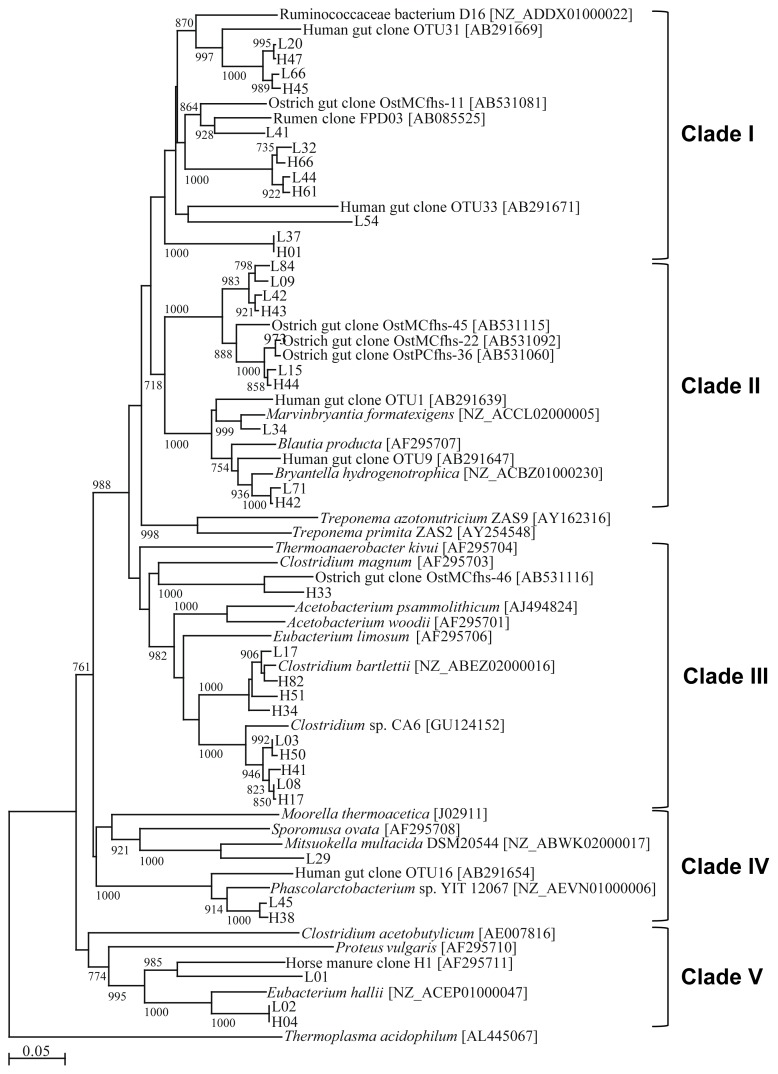
Phylogenetic tree based on formyltetrahydrofolate synthetase (*FHS*) sequences recovered from pig feces. The accession number of each sequence is shown in brackets. The scale bar represents 0.05 substitutions per amino acid position. Bootstrap values based on 1,000 trees are shown at each node; only values ≥700 are shown. Sequence names are the representative clone name for each operational taxonomic unit.

A phylogenetic tree was constructed using the deduced *FHS* amino acid sequences recovered in our study and *FHS* sequences in the databases (Figure 1). The OTUs were classified into 5 clades according to their phylogenetic positioning.

Table 2 summarizes the sequence similarities of OTUs rela tive to the *FHS* sequences deposited in the public databases and the HS scores calculated by Henderson et al [24]. Clade I consists of 5 OTUs (12 clones) isolated from animals in the HI group and 7 OTUs (14 clones) isolated from animals in the LI group (Table 2). Five OTUs (H45 and H47 and L20, L54, and L66) showed high similarity to the *FHS* sequence of *Ruminococcusceaea* bacterium D16; however, L54 showed lower similarity and was, thus, likely only distantly related to the *FHS* of *Ruminococcusceaea* bacterium D16. L41 showed the highest similarity and was closely related to the ostrich gut clone OstMCfhs-11. H44, H61, L32, and L44 also exhibited high similarities to OstMCfhs-11. Three OTUs (7 clones) isolated from animals in the HI treatment and 6 OTUs (13 clones) isolated from animals in the LI treatment were classified into clade II. L34 showed high similarity to the authentic acetogen, *Marvinbryantia formatexigens* (97%). H42 and L71 also exhibited high similarities to the authentic acetogen, *Bryantella hydrogenotrophica* (97% and 96%). H44 and L15 exhibited high similarities to the ostrich gut clones OstPCfhs-36 (99%) and OstMCfhs-22 (98%). The rest of the OTUs (H43, L09, L42, and L84) were the most similar to the ostrich clone OstMCfhs-45. Clade III consisted of 7 OTUs isolated from animals in the HI group and 3 from animals in the LI group. Twenty-three clones were isolated from animals in the HI group, almost double the number of clones isolated from animals in the LI group. H33 exhibited similarity to the ostrich gut clone OstMCfhs-46. Three OTUs, H34, H51, and H82 (5 clones) isolated from animals in the HI group and 1 OTU, L17 (1 clone), isolated from animals in the LI group showed high similarity to *FHS* from *Clostridium bartlettii* (96% to 98%). Three OTUs, H17, H41, and H50 (15 clones) isolated from animals in the HI group and 2 OTUs, L03 and L08 (8 clones), isolated from animals in the LI group exhibited relatively high similarities to *Clostridium* sp. CA6 (93% to 95%). Both clade IV and V contained only 3 OTUs. L29 from clade IV showed similarity to the *FHS* sequence of *Mitsuokella multacida*. Two other OTUs showed similarity to the *FHS* sequence of *Phascolarctobacterium* sp. YIT 12067. In clade V, all OTUs showed similarity to the *FHS* sequence of *Eubacterium hallii*. The HS scores of all OTUs in this clade were lower than 80%. L01, L02, and H04 contained 5, 7 (a total of 12), and 7 clones, respectively. Therefore, (12/50)×100 = 24% of clones isolated from animals in the LI group and (7/50)×100 = 14% of clones isolated from animals in the HI group were not authentic acetogens.

### Real-time polymerase chain reaction assays

**Figure 2:**
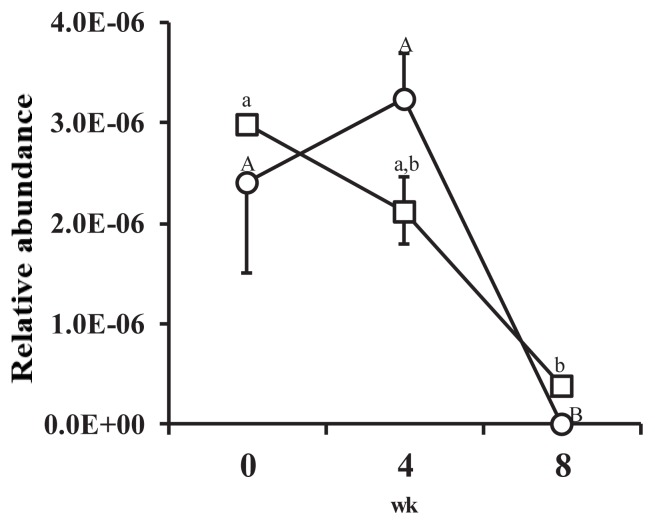
Changes in the relative abundance of formyltetrahydrofolate synthetase (*FHS*) genes against 16S ribosomal RNA genes from all bacteria recovered from porcine feces over the course of the experiment. Figure legend: ○, high-intake diet; □, low-intake diet. Error bar shows standard error (n = 4). ^A,B^ Letters indicate significant differences between mean values for each week in the high-intake treatment (p<0.05). ^a,b^ Letters indicate significant differences between mean values for each week in the low-intake treatment (p<0.05).

The proportion of *FHS* against 16S rDNA copy number was very low (Figure 2). The proportion of *FHS* in the feces of animals in the HI group increased at wk 4 but had significantly decreased by the 8th wk (p<0.05). The proportion of *FHS* in the feces of animals in the LI group continuously decreased from the start to the end of the experiment. The proportion of *FHS* at the 8th week was significantly lower than the proportion at wk 0 and wk 4 (p<0.05). During the experimental period, no significant differences in the proportion of *FHS* between treatments were observed.

## DISCUSSION

To date, *FHS* clone library analyses of the bovine rumen [13, 24], human large intestine [7], Tammar wallaby forestomach [13], and ostrich ceca [15], have been performed. However, there is no existing report on *FHS* gene diversity in the large intestine of pigs. This is, thus, the first reported molecular diversity analysis of homoacetogens in the pig large intestine.

A wide variety of *FHS* gene fragments were recovered from pig feces. Large numbers of *FHS* clones from unknown bacteria were recovered from the bovine rumen [14,24], human large intestine [7], Tammar wallaby forestomach [13], and ostrich ceca [15]. Similarly, the libraries derived from pig fecal samples in this study also contained a number of *FHS* sequences from unknown bacteria. Therefore, the GIT is probably colonized by many uncultivated acetogens. In the large intestines of pigs, methanogen diversities in the HI and LI groups was significantly different [10]. Unexpectedly, however, feed intake level did not affect acetogen diversity. This suggests that acetogen diversity in the large intestines of pigs was not related to host animal energy homeostasis.

The *FHS* clone libraries recovered in this study may contain *FHS* sequences from non-acetogenic bacteria [13,14]. The isolation and characterization of acetogenic bacteria is, thus, required to fully understand the diversity and function of acetogens in the pig large intestine.

The proportion of *FHS* decreased as pigs gained weight in both treatments (Figure 2). The exact reason for this is unclear, but the results suggest that the internal environment of the large intestine became increasingly hostile to acetogens as the animals gained weight. The proportion of *FHS* was also very low, and energy level had no significant effect on the proportion of *FHS*, suggesting that acetogen population density was also not affected by host energy homeostasis.

Finally, the same primer set was used in both the quan tification of *FHS* and in diversity analyses. According to the results of the *FHS* diversity analysis, 24% of all amplified *FHS* fragments of in LI and 14% in HI, not all *FHS* fragments amplified with primer set used in this study were authentic acetogens. Therefore, the values obtained from the real-time PCR may have been somewhat overestimated.

Further study is required to clarify the reason the reduc tion of population density of acetogens during fattening period.

## Figures and Tables

**Table 1:** Ingredients and chemical compositions of diets

Items	
Ingredients (%)	
Maize	69.1
Wheat bran	10.0
Soybean meal	10.0
Rapeseed meal	5.0
Defatted rice bran	3.0
Fish meal	1.0
Dicalcium phosphate	0.15
Calcium carbonate	0.93
Salt	0.02
Vitamin B complex	0.02
Trace minerals	0.02
Chemical composition	
DE (MJ/kg)	3.36
TDN (%)	76.2
Crude protein (%)	13.8
Crude fiber (%)	4.47

DE, digestible energy; TDN, total digestible nutrients.

**Table 2:** Blast search results and homoacetogen similarity (HS) score of amino acid sequences and operational taxonomic units (OTU) derived from 2 *FHS* clone libraries derived from pig fecal material

Clade	OTU1)	No. of clone	Closest relative (Accession No.)	Similarity (%)	HS score2)
I	H45	1	*Ruminococcaceae* bacterium D16 (NZ_ADDX01000022)	86	92.5
	H47	1	*Ruminococcaceae* bacterium D16 (NZ_ADDX01000022)	87	95.0
	L20	5	*Ruminococcaceae* bacterium D16 (NZ_ADDX01000022)	87	95.0
	L66	1	*Ruminococcaceae* bacterium D16 (NZ_ADDX01000022)	86	90.0
	L54	2	*Ruminococcaceae* bacterium D16 (NZ_ADDX01000022)	76	82.5
	H61	3	Ostrich gut clone OstMCfhs-11 (AB531081)	84	90.0
	H66	1	Ostrich gut clone OstMCfhs-11 (AB531081)	85	90.0
	L32	2	Ostrich gut clone OstMCfhs-11 (AB531081)	85	90.0
	L41	1	Ostrich gut clone OstMCfhs-11 (AB531081)	89	97.5
	L44	2	Ostrich gut clone OstMCfhs-11 (AB531081)	84	90.0
	H01	6	Ostrich gut clone OstMCfhs-11 (AB531081)	84	90.0
	L37	1	Ostrich gut clone OstMCfhs-11 (AB531081)	84	90.0
II	H43	1	Ostrich gut clone OstMCfhs-45 (AB531115)	93	95.0
	L09	3	Ostrich gut clone OstMCfhs-45 (AB531115)	93	95.0
	L42	1	Ostrich gut clone OstMCfhs-45 (AB531115)	92	92.5
	L84	3	Ostrich gut clone OstMCfhs-45 (AB531115)	94	95.0
	H44	4	Ostrich gut clone OstPCfhs-36 (AB531060)	99	97.5
	L15	4	Ostrich gut clone OstMCfhs-22 (AB531092)	98	97.5
	L34	1	M*arvinbryantia formatexigens* DSM 14469 (NZ_ACCL02000005)	97	100.0
	H42	2	*Bryantella hydrogenotrophica* DSM 10507 (NZ_ACBZ01000230)	97	100.0
	L71	1	*Bryantella hydrogenotrophica* DSM 10507 (NZ_ACBZ01000230)	96	100.0
III	H33	3	Ostrich gut clone OstMCfhs-46 (AB531116)	93	87.5
	H34	3	*Clostridium bartlettii* DSM 16795 (NZ_ABEZ02000016)	96	95.0
	H51	1	*Clostridium bartlettii* DSM 16795 (NZ_ABEZ02000016)	96	92.5
	H82	1	*Clostridium bartlettii* DSM 16795 (NZ_ABEZ02000016)	98	90.0
	L17	1	*Clostridium bartlettii* DSM 16795 (NZ_ABEZ02000016)	98	90.0
	H17	9	*Clostridium* sp. CA6 (GU124152)	95	90.0
	H41	5	*Clostridium* sp. CA6 (GU124152)	95	90.0
	H50	1	*Clostridium* sp. CA6 (GU124152)	93	87.5
	L03	2	*Clostridium* sp. CA6 (GU124152)	94	90.0
	L08	6	*Clostridium* sp. CA6 (GU124152)	95	90.0
IV	L29	1	*Mitsuokella multacida* DSM 20544 (NZ_ABWK02000017)	88	95.0
	H38	1	*Phascolarctobacterium* sp. YIT 12067 (NZ_AEVN01000006)	93	80.0
	L45	1	*Phascolarctobacterium* sp. YIT 12067 (NZ_AEVN01000006)	93	82.5
V	L01	5	*Eubacterium hallii* DSM 3353 (ACEP01000047)	74	70.0
	H04	7	*Eubacterium hallii* DSM 3353 (ACEP01000047)	90	65.0
	L02	7	*Eubacterium hallii* DSM 3353 (ACEP01000047)	90	65.0

1) OTU names starting with “H” and “L” were derived from the high-intake library and a low-intake library, respectively. OTU names are representative of the clone name of each OTU.

2) HS score was calculated according to the methods of Henderson et al [24].
